# Rotavirus gastroenteritis in Indian children < 5 years hospitalized for diarrhoea, 2012 to 2016

**DOI:** 10.1186/s12889-019-6406-0

**Published:** 2019-01-15

**Authors:** Sidhartha Giri, Nayana P. Nair, Ann Mathew, B. Manohar, Anna Simon, Tejinder Singh, S. Suresh Kumar, M. A. Mathew, Sudhir Babji, Rashmi Arora, C. P. Girish Kumar, S. Venkatasubramanian, Sanjay Mehendale, Mohan D. Gupte, Gagandeep Kang

**Affiliations:** 1 Division of Gastrointestinal Sciences, Christian Medical College, Vellore, Tamil Nadu India; 2 Department of Paediatrics, St. Stephen’s Hospital, Tis Hazari, New Delhi India; 3 Department of Paediatrics, SV Medical College, Tirupati, Andhra Pradesh India; 4 Department of Paediatrics, Christian Medical College, Vellore, Tamil Nadu India; 5 Department of Paediatrics, Christian Medical College, Ludhiana, Punjab India; 6 Punjagutta, Pragna Hospital, Hyderabad, Telangana India; 7 Department of Paediatrics, Malankara Orthodox Syrian Church Medical College, Kolenchery, Kerala India; 8 Indian Council of Medical Research, New Delhi, India; 9 National Institute of Epidemiology, Chennai, Tamil Nadu India; 10 Present address: Translational Health Science and Technology Institute (THSTI), Faridabad, India

**Keywords:** Diarrhoea, Gastroenteritis, Enzyme immunoassay, Genotypes, India, Polymerase chain reaction, Rotavirus

## Abstract

**Background:**

In 2016, the Government of India introduced the oral rotavirus vaccine (ROTAVAC, Bharat Biotech, India) in 4 states of India as part of the Universal Immunization Programme, and expanded to 5 more states in 2017. We report four years of data on rotavirus gastroenteritis in hospitalized children < 5 years of age prior to vaccine introduction.

**Methods:**

Children from 7 sites in southern and northern India hospitalized for diarrhoea were recruited between July 2012 and June 2016. Stool samples were screened for rotavirus using enzyme immunoassay (EIA). The EIA positive samples were genotyped by reverse-transcription polymerase chain reaction.

**Results:**

Of the 5834 samples from the 7 sites, 2069 (35.5%) were positive for rotavirus by EIA. Genotyping was performed for 2010 (97.1%) samples. G1P[8](56.3%), G2P[4](9.1%), G9P[4](7.6%), G9P[8](4.2%), and G12P[6](3.7%) were the common genotypes in southern India and G1P[8](36%), G9P[4](11.4%), G2P[4](11.2%), G12P[6](8.4%), and G3P[8](5.9%) in northern India.

**Conclusions:**

The study highlights the high prevalence of rotavirus gastroenteritis in India and the diversity of rotavirus genotypes across different geographical regions. Pre- vaccine surveillance data is necessary to evaluate the potential change in admission rates for gastroenteritis and circulating rotavirus genotypes after vaccine introduction, thus assessing impact.

**Electronic supplementary material:**

The online version of this article (10.1186/s12889-019-6406-0) contains supplementary material, which is available to authorized users.

## Background

Rotavirus is a genus of the *Reoviridae* family, and is classified into 7 groups (A-G) [1]. Of the seven groups, group A rotavirus is the most important cause of severe acute diarrhoea in infants and young children globally [1]. Rotavirus consists of 11 segments of double stranded RNA, surrounded by 3 layers of proteins. The outer capsid consists of VP7 (G type) and VP4 (P type) proteins, which are essential for the binary system of rotavirus classification [1]. The four common G types (G1-G4), along with G9 and G12, in association with P[6] and P[8], comprise the common circulating rotavirus genotypes globally [1].

Rotavirus causes an estimated 11.37 million episodes of acute gastroenteritis (AGE) in children < 5 years annually in India, requiring 3.27 million outpatient visits and 872,000 inpatient admissions resulting in total direct costs of Indian Rupee (INR) 10.37 billion per year [2]. In 2011, it is estimated that there were 78,000 deaths due to rotavirus associated AGE in India, with the majority (75.6%, 59,000) in the first 2 years in life [2].

The Indian Rotavirus Strain Surveillance Network, comprising of 10 representative hospitals from 7 Indian cities, estimated rotavirus to cause 40% of hospitalizations due to AGE in children < 5 years during 2005–2009 [3]. The study demonstrated regional trends in rotavirus strain diversity along with the emergence of new strains [3]. A few other prospective hospital based surveillance studies in < 5 years children, conducted between 2009 to 2012, found rotavirus to be associated with approximately 26–39% of AGE cases [4, 5]. The climate in north India varies from that in south India. The northern sites have more temperate climate, with a substantial fall in temperature during the winter months. In contrast, south India has a tropical climate. A difference in the seasonal trend in rotavirus associated diarrhoea across northern and southern sites in India has been reported in a few surveillance studies from India [4, 6].

For the prevention of rotavirus associated AGE, two oral rotavirus vaccines, Rotarix (RV1; monovalent G1P[8]; GlaxoSmithKline Biologicals, Belgium) and RotaTeq (RV5; pentavalent G1, G2, G3, G4, P[8], Merck Vaccines, NJ, USA), have been commercially available in India since 2006 [7]. In 2015, the ROTAVAC vaccine (Bharat Biotech, India), containing the live 116E rotavirus strain (G9P[11]), was introduced as the first indigenously developed vaccine at a substantially lower price [8]. In April 2016, the vaccine was introduced into the Universal Immunization Programme (UIP) by the Government of India in 4 states (Andhra Pradesh, Haryana, Himachal Pradesh, Odisha), with the subsequent introduction in 5 additional states by September 2017 (Rajasthan, Madhya Pradesh, Assam, Tripura, Tamil Nadu).

Pre- vaccine surveillance data is crucial to study the potential change in admission rates for gastroenteritis and circulating rotavirus genotypes after vaccine introduction. We report the findings of a multicentre hospital-based surveillance from July 2012 to June 2016 from 7 Indian sites on the clinical, epidemiological, and virological features of severe rotavirus disease among Indian children < 5 years of age, with the use of standardized protocols for enrolment and diagnostic evaluation.

## Methods

### Study sites

The study was conducted in hospitals in 7 cities of India, with 5 sites in southern India (Vellore, Trichy, Kolenchery, Hyderabad, Tirupati) and 2 in the north (Delhi, Ludhiana). The hospitals included Christian Medical College (CMC, Vellore), Child Jesus Hospital (Trichy), Malankara Orthodox Syrian Church Medical College (Kolenchery), Pragna Hospital (Hyderabad), SV Medical College (Tirupati) in the south, and Christian Medical College (Ludhiana), St. Stephen’s Hospital (New Delhi) in the north. These hospitals are tertiary-level health care centres providing both general and specialized medical care. All the testing was performed at CMC, Vellore, using a modification of the World Health Organization (WHO) generic protocol for rotavirus surveillance [9]. Administrative and technical coordination was performed by the Indian Council of Medical Research (ICMR) and the National Institute of Epidemiology (NIE).

### Enrolment criteria

Children ≤59 months of age hospitalized for at least 6 h for watery diarrhoea, and given supervised oral, or intravenous, rehydration, were eligible for enrolment. All the eligible children were enrolled after obtaining informed consent from the parents/guardian. An episode of AGE was defined as ≥3 loose stools over a 24 h period. Eligible patients were recruited if they provided a stool sample within 48 h of admission to rule out nosocomial infection. The exclusion criteria for the study were: children > 59 months of age, duration of diarrhoea > 5 days, blood in stool (dysentery), or < 3 episodes in a 24 h period.

### Clinical assessment

Detailed clinical information on onset of diarrhoea, vomiting, fever, and dehydration were collected. The information included duration and number of diarrhoeal and/or vomiting episodes, degree of fever, severity of dehydration, and treatment. The Vesikari scoring system was used to assess the severity of diarrhoeal episodes (0–5 for mild, 6–10 for moderate, 11–15 for severe, and 16–20 for very severe) [10].

### Sample collection and transport

One diarrhoeal stool sample was collected from each enrolled child. All stool samples were either transported to the testing laboratory within two hours of collection or stored at the sites at 4 °C till transport. The stool samples from the sites other than Vellore were transported once every month in boxes with ice packs. On reaching the laboratory, the samples were tested for rotavirus antigen on the day of receipt, and aliquots of samples were stored at -70 °C till further testing.

### Laboratory procedures

A commercial enzyme immunoassay (EIA) was used to screen all the stool samples for rotavirus antigen (Rotaclone; Meridian Bioscience, Inc.). All EIA positive samples were further tested for VP7 (G type) and VP4 (P type) genes using reverse-transcription polymerase chain reaction (RT-PCR) according to the guidelines of the World Health Organization (WHO) for rotavirus detection and characterization [11]. In brief, viral RNA extraction was performed from 20% stool suspension (*W*/*V*) using the QIAamp Viral RNA Mini Kit (Qiagen). This was followed by reverse transcription for forming complementary DNA (cDNA) using Moloney murine reverse transriptase enzyme (Superscript II MMLV-RT, Invitrogen) and random primers (Invitrogen). The cDNA was used as the template for genotyping in a hemi-nested multiplex PCR for VP7 and VP4 genes, using published oligonucleotide primers to identify VP7 genotypes G1, G2, G3, G4, G8, G9, G10, G12, and VP4 genotypes P[4], P[6], P[8], P[9], P[10], and P[11] [3, 6, 12, 13]. For samples negative by genotyping PCR, a VP6 gene specific PCR was performed to confirm rotavirus positivity [3, 6]. Sanger sequencing was used to evaluate strains that could not be typed [3, 6].

### Statistical analysis

All sites submitted summary data on all diarrhoea-related hospital admissions. The forms were scrutinized for completeness, and the data were entered into Excel 2003 (Microsoft). The data were analyzed to evaluate the prevalence of rotavirus associated diarrhoea, genotype diversity, as well as temporal and regional variation in rotavirus strain prevalence from 2012 to 2016. To evaluate the prevalence of rotavirus associated diarrhoea, the proportion of rotavirus positive stool samples out of the total samples collected was calculated. SPSS software (version 21, IBM) was used to calculate *p*- value using Fisher’s exact test. A *p* value < 0.05 was considered to be statistically significant. For evaluating the regional variation in rotavirus positivity, the month-wise proportion of rotavirus positivity by EIA in the 2 north Indian sites and 5 south Indian sites were compared over the study period of 4 years.

### Ethics

The study was approved by the institutional review board of CMC, Vellore, India (IRB Minute. No. 7311 dated 20.10.2010). The study was also approved by the institutional review boards/ethics committees of all the sites, which included St. Stephen’s Hospital (New Delhi), SV Medical College (Tirupati), Christian Medical College (Ludhiana), Pragna hospital (Hyderabad), Child Jesus Hospital (Trichy), and Malankara Orthodox Syrian Church Medical College (Kolenchery). All the study participants were enrolled after obtaining written informed consent from the parents/guardian.

## Results

### Site wise hospitalization due to rotavirus associated diarrhoea

**Fig. 1:**
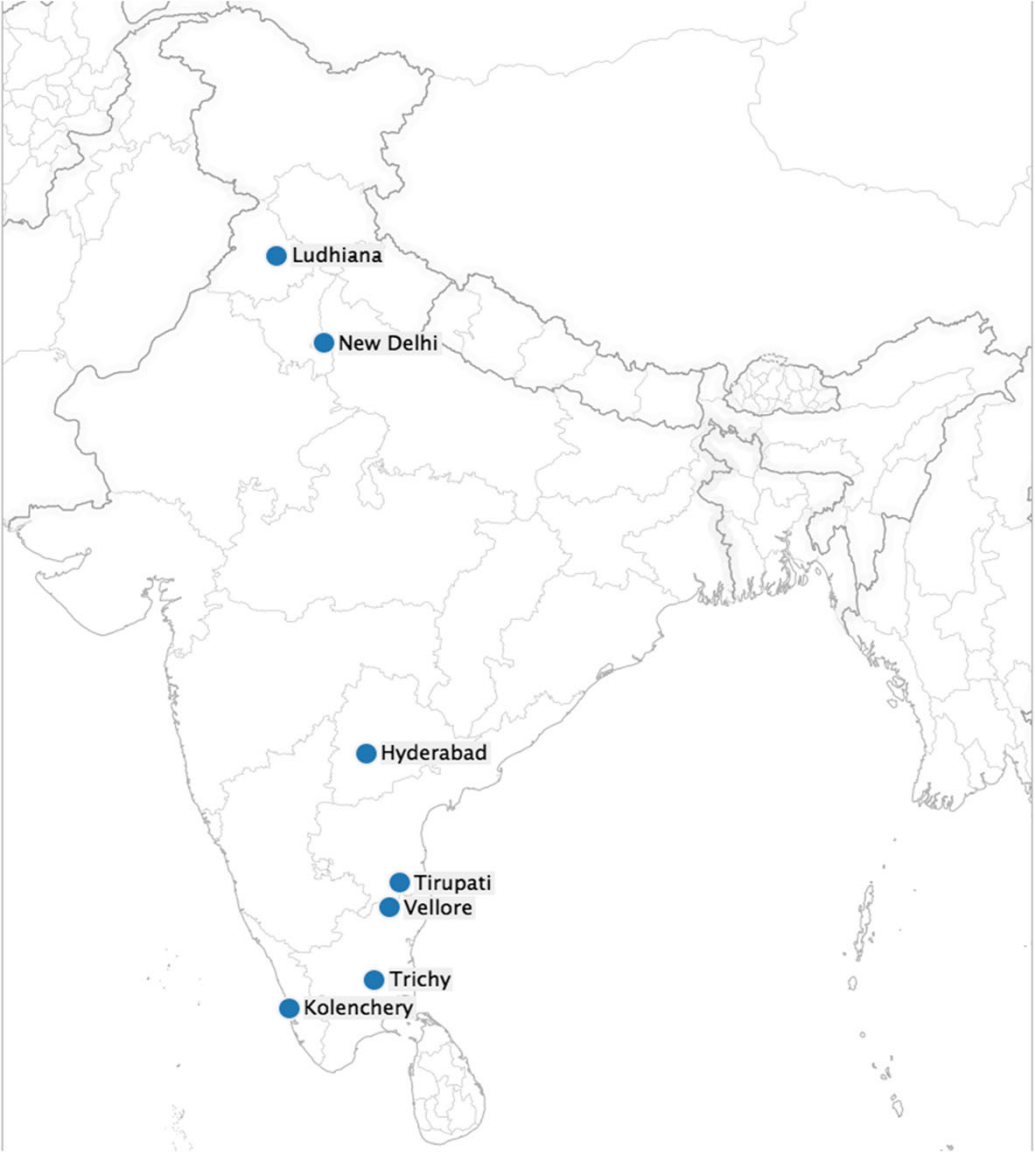
Seven sites in the rotavirus strain surveillance network from 2012 to 2016

During the four years of surveillance (July 2012–June 2016), 6576 eligible children hospitalized for AGE were enrolled at the 7 sites (Fig. 1, Table 1). A total of 5834 children (88.7%) were included in the final analysis, while 742 (11.3%) were excluded (Additional file 1: Table S1). Stool samples of 35.5% (2069/5834) patients were positive for rotavirus by enzyme immunoassay (EIA). The overall rotavirus positivity was 41.7% (663/1590) in the north compared to 33.1% (1406/4244) in the south (Table 1). The detection rate of rotavirus ranged from 23.5% in Tirupati to 49.4% in Trichy (Table 1).

**Table 1:** Hospital based surveillance of rotavirus associated diarrhoea in children <5 years in India

Site	Number of children enrolled	Number of children excluded	Number of children in final analysis	ELISA positive for rotavirus
Vellore	1403	66 (4.7%)	1337 (95.3%)	392 (29.3%, 392/1337))
Kolenchery	1109	210 (18.9%)	899 (81.1%)	389 (43.3%, 389/899)
Trichy	624	63 (10.1%)	561 (89.9%)	277 (49.4%, 277/561)
Ludhiana	696	127 (18.2%)	569 (81.8%)	213 (37.4%, 213/569)
Hyderabad	670	7 (1%)	663 (99%)	164 (24.7%, 164/663)
New Delhi	1258	237 (18.8%)	1021 (81.2%)	450 (44.1%, 450/1021)
Tirupati	816	32 (3.9%)	784 (96.1%)	184 (23.5%, 184/784)
Total	6576	742 (11.3%)	5834 (88.7%)	2069 (35.5%, 2069/5834)

The north Indian sites include New Delhi and Ludhiana. The south Indian sites include Vellore, Kolenchery, Trichy, Hyderabad, and Tirupati

**Fig. 2:**
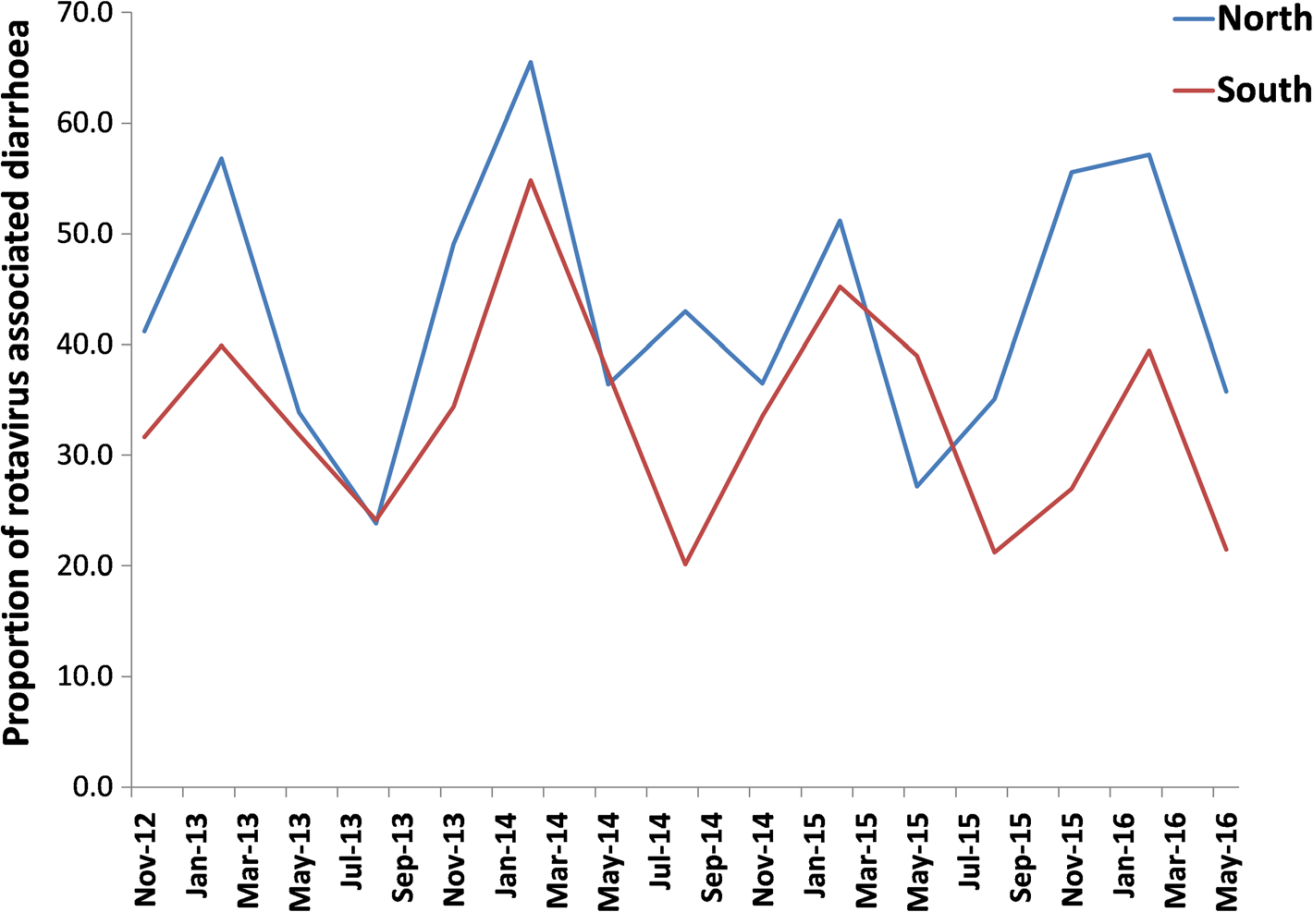
Temporal distribution of rotavirus-positive cases in northern (2 sites) and southern (5 sites) regions of India

Rotavirus-associated diarrhoea was seen throughout the year at all the sites. However, there was distinct seasonality for rotavirus associated diarrhoea, with highest prevalence seen during December–February (Fig. 2).

### Characteristics of rotavirus-infected and uninfected children hospitalized with diarrhoea

We evaluated the demographic characteristics of children hospitalized with diarrhoea (Table 2). There was no significant difference in the mean age (± standard deviation, SD) of children hospitalized with rotavirus-associated diarrhoea (15.0 ± 11.2 months), compared to rotavirus negative cases (14.9 ± 12.5 months). Children hospitalized with rotavirus associated gastroenteritis had more severe disease than children with rotavirus-negative diarrhoea (Table 2). Children with rotavirus associated diarrhoea had significantly higher vomiting (76.1% versus 66.2%) than rotavirus negative cases (Table 2). Infants (0–11 months) contributed the major proportion (47.9%) of rotavirus associated diarrhoea, followed by children aged 12 to 23 months (33.5%) and children ≥2 years (18.6%) (Table 2).

**Table 2:** Characteristics of rotavirus infected and uninfected children hospitalized with acute gastroenteritis

Variable	Rotavirus positive (*N* = 2069)	Rotavirus negative (*N* = 3765)	*p* value
Male	1304 (63.0%)	2339 (62.1%)	0.52
Age, mean months ± SD	15.02 ± 11.2	14.93 ± 12.5	0.8
Age group distribution			
< 1 year (0–11 months)	992 (47.9%)	1924 (51.1%)	0.02
1 year to < 2 years (12–23 months)	693 (33.5%)	1051 (27.9%)	< 0.001
2 years to <5 years (24–59 months)	384 (18.6%)	790 (21%)	0.03
Duration of hospital stay in days, mean ± SD	3.2 ± 2.2	3.4 ± 2.9	< 0.001
Temperature	1273 (61.5%)	2360 (62.7%)	0.38
Vomiting	1574 (76.1%)	2490 (66.15%)	< 0.001
Vesikari score, mean ± SD	11.1 ± 2.8	10.6 ± 3.0	< 0.001
Disease severity by Vesikari score			
Mild (1–5)	83 (4.01%)	186 (4.94%)	0.11
Moderate (6–10)	685 (33.1%)	1532 (40.6%)	< 0.001
Severe (11–15)	1251 (60.4%)	1947 (51.7%)	< 0.001
Very severe (16–20)	50 (2.4%)	100 (2.6%)	0.58

### G and P typing at 7 surveillance sites from 2012 to 2016

Of the 2069 samples that were positive for rotavirus by EIA, genotyping was performed for 2010 strains (97.1%); of which 1960 (97.5%) were G typed, and 1955 were P typed. Both G and P types were determined for 1947 samples (Table 3). The most prevalent G and P type combinations were G1P[8] (1002 strains [49.9%]), G2P[4] (196 [9.8%]), G9P[4] (177 [8.8%]) and G12P[6] (105 strains [5.2%]) (Table 4). A number of other genotypes, such as G12P[8], G1P[4], G1P[6], G2P[6], G3P[8], G9P[8], and G9P[6], were seen at all surveillance sites at lower proportions (Table 3). Genotypes that were identified only rarely were G2P[8], G3P[4], G3P[6], G4P[6], G12P[11], and G12P[4] (Table 3). We did not find any significant association between the common rotavirus genotypes and severity of diarrhoea (Additional file 2: Table S2).

**Table 3:** Genotype distribution at the seven surveillance sites in India during the 4 years study period

	Vellore		Kolenchery		Trichy		Ludhiana		Hyderabad		New Delhi		Tirupati		Total	
	N	%	N	%	N	%	N	%	N	%	N	%	N	%	N	%
G1P[4]	2	0.5	4	1.0	3	1.1	–	–	2	1.3	4	0.9	3	1.7	18	0.9
G1P[6]	7	1.8	3	0.8	8	2.9	3	1.5	1	0.7	21	4.8	2	1.1	45	2.2
G1P[8]	238	62.6	189	49.3	179	65.6	54	26.6	64	41.8	177	40.4	101	56.1	1002	49.9
G2P[4]	32	8.4	21	5.5	23	8.4	37	18.2	36	23.5	35	8.0	12	6.7	196	9.8
G2P[6]	–	–	5	1.3	–	–	5	2.5	–	–	12	2.7	1	0.6	23	1.1
G2P[8]	1	0.3	–	–	–	–	–	–	–	–	–	–	–	–	1	0.05
G3P[4]	–	–	1	0.3	–	–	–	–	–	–	2	0.5	1	0.6	4	0.2
G3P[6]	–	–	1	0.3	–	–	–	–	–	–	–	–	–	–	1	0.05
G3P[8]	4	1.1	2	0.5	9	3.3	11	5.4	3	2.0	27	6.2	2	1.1	58	2.9
G4P[6]	–	–	1	0.3	–	–	–	–	–	–	–	–	–	–	1	0.05
G9P[4]	31	8.2	25	6.5	16	5.9	32	15.8	9	5.9	41	9.4	23	12.8	177	8.8
G9P[6]	1	0.3	3	0.8	–	–	11	5.4	–	–	4	0.9	–	–	19	0.9
G9P[8]	6	1.6	39	10.2	9	3.3	9	4.4	2	1.3	5	1.1	1	0.6	71	3.5
G10P[11]	9	2.4	1	0.3	–	–	–	–	–	–	–	–	–	–	10	0.5
G12P[4]	–	–	2	0.5	–	–	2	1.0	–	–	2	0.5	1	0.6	7	0.3
G12P[6]	10	2.6	31	8.1	2	0.7	11	5.4	–	–	43	9.8	8	4.4	105	5.2
G12P[8]	10	2.6	8	2.1	3	1.1	3	1.5	6	3.9	10	2.3	9	5.0	49	2.4
G12P[11]	–	–	–	–	–	–	–	–	–	–	1	0.2	–	–	1	0.05
Mixed	18	4.7	38	9.9	15	5.5	23	11.3	11	7.2	45	10.3	9	5.0	159	7.9
Partially typed	4	1.1	2	0.5	3	1.1	–	–	7	4.6	3	0.7	2	1.1	21	1.0
Untyped	7	1.8	7	1.8	3	1.1	2	1.0	12	7.8	6	1.4	5	2.8	42	2.1
Total	380		383		273		203		153		438		180		2010	100.0

N = number of genotyped samples, % (Percentage)

**Table 4:** Surveillance studies on rotavirus-associated diarrhoea in hospitalized children < 5 years from India

Study site/s	Type of study	Study period	Age group	Number of stool samples	Common rotavirus genotypes	Reference
Lucknow	Single centre, cross-sectional	September 2004–April 2008	1–36 months	412	G1P[8] (21.5%), G3P[6] (16.5%), G1P[6] (8.9%), G2P[8] (8.9%)	Mishra V, et al. [19]
Andaman & Nictobar islands	Multicentre, cross-sectional	October 2010–February 2012	6–60 months	296	G2P[4] (44.7%), G1P[8] (25.5%), G9P[4] (21.2%), G1P[4] (4.3%)	Reesu R, et al. [20]
Kerala	Multicentre, cross-sectional	February 2009–January 2011	<5 years	1807	G1P[8] (49.7%), G9P[8] (26.4%), G2P[4] (5.5%), G9P[4] (2.6%), G12P[6] (1.3%)	Mathew MA, et al. [21]
Kolkata	Multicentre, cross-sectional	January 2011–December 2013	≤5 years	830	G2P[4] (31.6%), G9P[4] (27.5%), G1P[8] (13.5%), G9P[8] (12%), G12P[8] (2.7%)	Mullick S, et al. [22]
Delhi	Multicentre, cross-sectional	August 2007–July 2012	<5 years	756	G12P[6] (10%), G1P[8] (7.2%), G2P[4] (7.2%), G9P[4] (6.5%), G9P[8] (5.2%)	Tiku VR, et al. [23]
Manipur	Single centre, cross-sectional	December 2005–March 2008	< 5 years	489	G1P[8] (36%), G2P[4] (22%), G12P[6] (8%), G1P[4] (3%), G9P[6] (3%)	Mukherjee A, et al. [24]
Delhi	Single centre, cross-sectional	February 2005–March 2007	< 2 years	862	G1P[8] (26%), G1P[4] (12%), G1P[6] (11%), G2P[4] (8%), G9P[8] (5%)	Chakravarti A, et al. [25]
12 medical schools across India	Multi centre, cross-sectional	April 2011–July 2012	≤59 months	2051	G1P[8] (23.8%), G2[P4] (12.9%), G9P[4] (8.1%), G12P[6] (6.8%), G9[P8] (5%)	Saluja T, et al. [5]
10 hospitals in 7 cities	Multicentre cross-sectional	November 2005–June 2009	≤59 months	7285	G1P[8] (18.8%), G2P[4] (16.9%), G9P[8] (6.1%), G12P[6] (5.6%), G12P[8] (3.2%)	Kang G, et al. [3]
3 hospitals in 3 cities	Multicentre cross-sectional	July 2009–June 2011	≤59 months	1191	G1P[8] (33.7%), G2P[4] (17.5%), G9P[4] (10.9%), G9P[8] (6.3%), G12P[6] (6.3%)	Babji S, et al. [4]
7 hospital in 7 cities	Multicentre cross-sectional	July, 2012-June, 2016	≤59 months	5834	G1P[8] (49.9%), G2P[4] (9.8%), G9P[4] (8.8%), G12P[6] (5.2%), G9P[8] (3.5%)	This study

G12 strains were seen in combination with P[4], P[6], P[8] and P[11], and comprised 8.1% (162) of the total strains genotyped. The highest proportion of G12 strains were identified in New Delhi (12.8%) in the north and Kolenchery (10.7%) in the south (Table 3). G3P[8] strains were identified from 2015 onwards and were significantly more common in the north (6.2% in New Delhi, 5.4% in Ludhiana) compared to southern sites (ranged from 0.5% in Kolenchery to 3.3% in Trichy) (*p* < 0.001). Mixed infections were approximately 8% of all the genotyped strains, while partially typed and untyped samples comprised 1 and 2.1% of the genotyped samples respectively (Table 3).

The year wise distribution of rotavirus strains showed interesting trends. G3P[8] appeared in 2015 (0.4%) and by 2016 the frequency increased to 12.1% (Additional file 3: Table S3). During 2015–2016, G9P[4] constituted 22.5% of the rotavirus genotypes, and the maximum cases were from New Delhi. The proportion of G1P[8] strains, which increased from 37.1% during 2012–2013 to 69.7% during 2014–2015, showed a sharp decrease during 2015–2016 (24.2% of total strains).

**Fig. 3:**
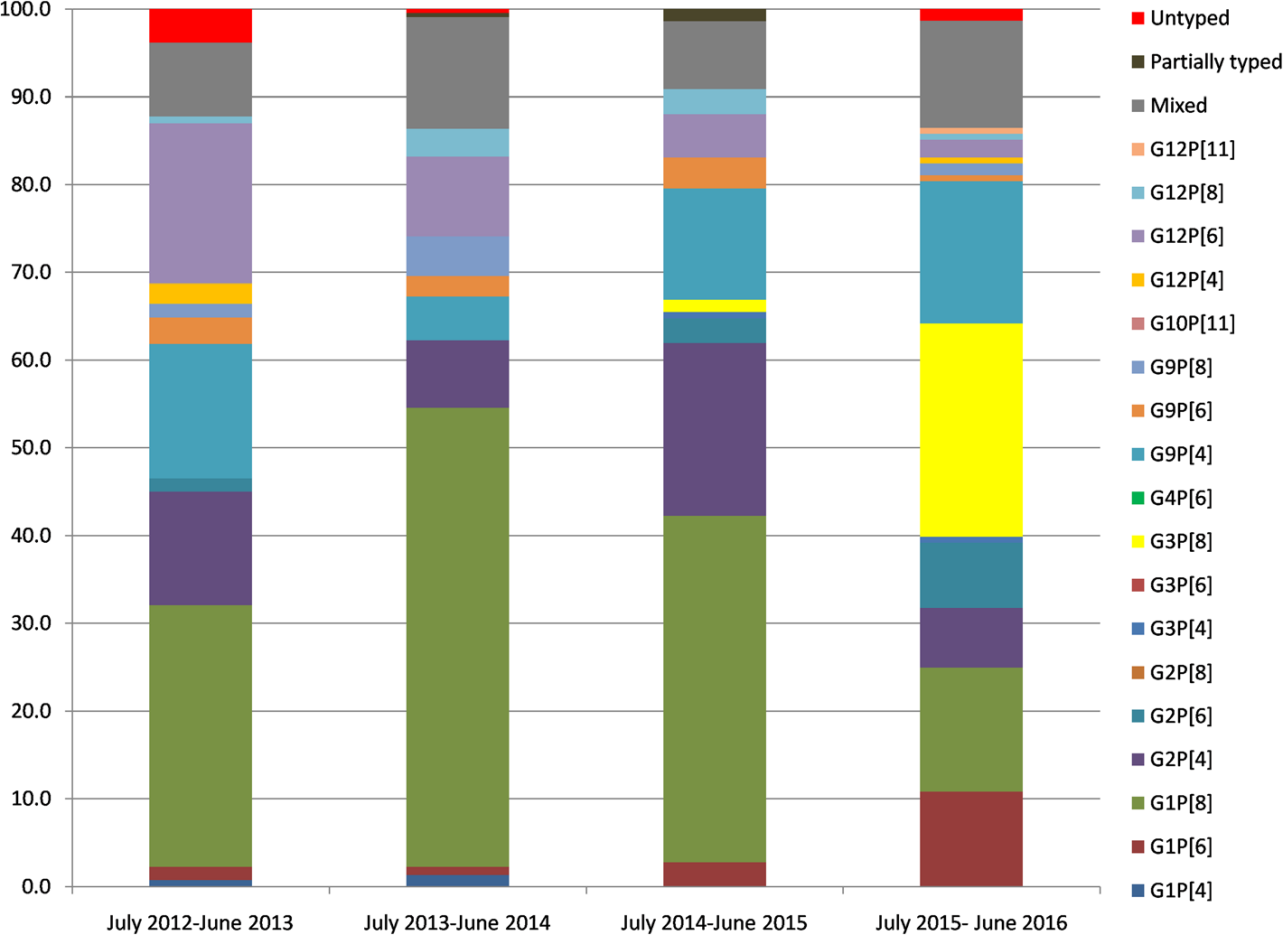
Distribution of rotavirus genotypes in < 5 years children admitted with acute gastroenteritis in north Indian sites

**Fig. 4:**
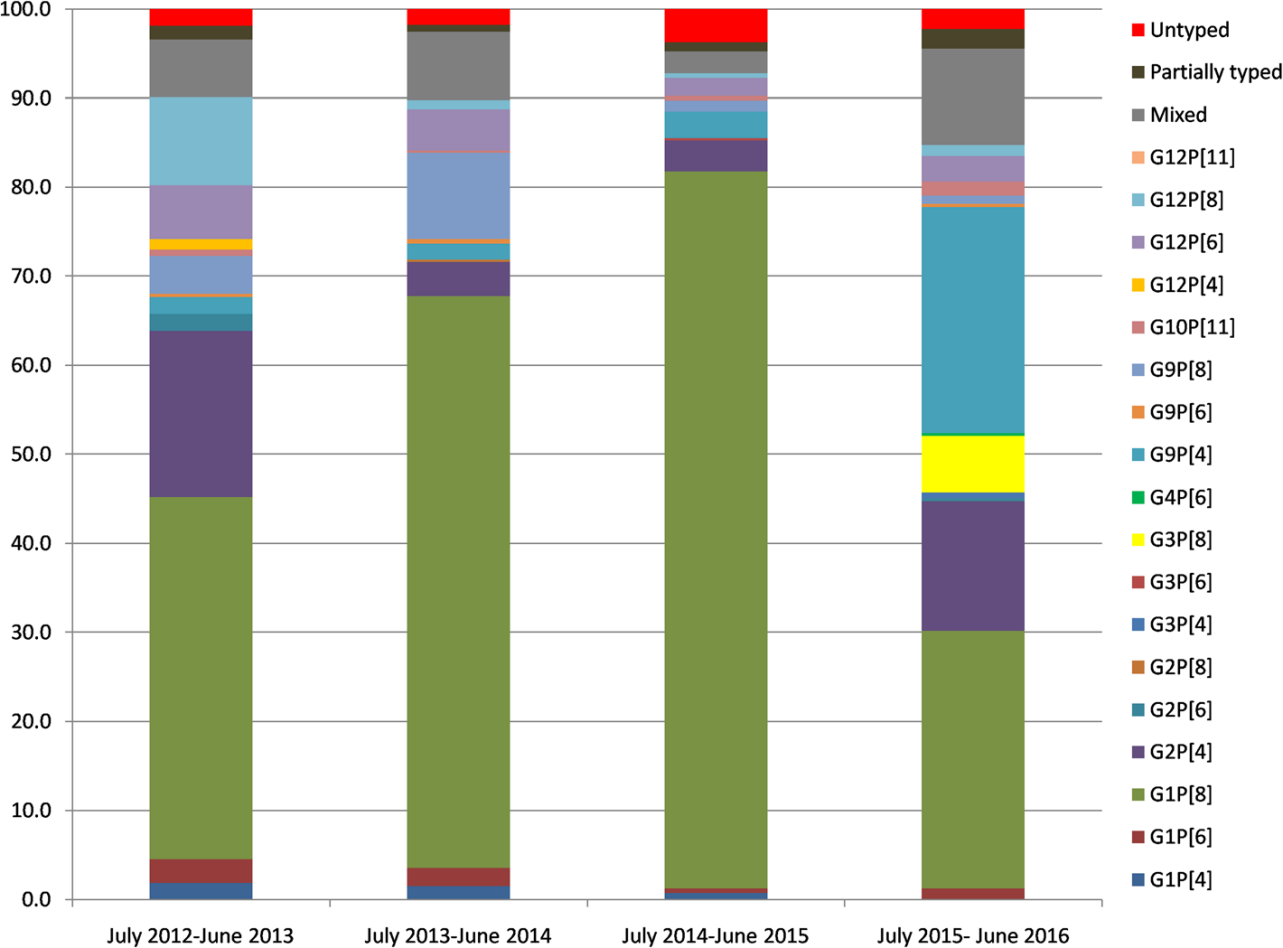
Distribution of rotavirus genotypes in < 5 years children admitted with acute gastroenteritis in south Indian sites

Figures 3 and 4 show the difference in year wise distribution of rotavirus genotypes in the northern and southern sites respectively. Overall, the common genotypes in the northern sites were G1P[8] (36%), G9P[4] (11.4%), G2P[4] (11.2%), G12P[6] (8.4%), and G3P[8] (5.9%) (Additional file 4: Table S4). Among the north Indian sites, G1P[8] strains increased from 29.8% during 2012–2013 to 52.3% in 2013–2014, but then decreased to 14.2% in 2016. G9P[4] strains decreased from 15.3% in 2012–2013 to 5% in 2014, but increased to 16.2% by 2016 (Fig. 3). Emergence of G3P[8] was noticed in 2015 and by 2016, the prevalence was 24.3% (Fig. 3). G2P[6] strains showed an increase (7.4%) by 2016 (Fig. 3) in northern India, whereas the prevalence came down in the south.

The common genotypes detected in the southern sites were G1P[8] (56.3%), G2P[4] (9.1%), G9P[4] (7.6%), G9P[8] (4.2%), and G12P[6] (3.7%) (Fig. 4). The south Indian sites showed a marked increase in G1P[8] strain from 2012 to 2015 (40.7 to 80.5%), but the prevalence came down in 2016 (28.9%) (Additional file 5: Table S5). Similar to north India, G3P[8] strains emerged in south India during the period of June 2015 to July 2016 with a prevalence was 6.3%. A rise in G9P[4] strains was noticed from 2012 to 2016 (1.9 to 25.4%). Uncommon genotype such as G2P[8] and G4P[6] were found in southern sites, but not in north Indian sites.

Mixed genotypes were more common in the north (10.6%) than the south (6.6%) (*p* = 0.003). The proportions of partially typed and untyped samples were significantly more common in the southern sites (1.3 and 2.5% in the south, compared to 0.5 and 1.2% in the north respectively) (*p* = 0.01).

## Discussion

This 4 year study emphasizes the role of rotavirus as a major cause for acute gastroenteritis in children < 5 years in India before the introduction of the oral rotavirus vaccine into the national immunization programme. In more than 5800 stool samples, rotavirus was detected in 35.5%, comparable to the 2005–2009 period when rotavirus was detected in stools of 40% of children hospitalized for diarrhoea [3] An earlier review of studies on rotavirus disease in India found approximately 34% (inter study variation: 19–50%) of all AGE related hospitalizations to be due to rotavirus infections [7].

The study also demonstrates the early prevalence of rotavirus gastroenteritis in India, with most disease in the first 2 years of life. Although the percentage of rotavirus-associated diarrhoea remained similar from 2012 to 2016, significant changes in rotavirus strain diversity were observed across all sites. G1P[8] (49.9%) and G2P[4] (9.8%) were the two most common genotypes in this study, which is similar to the previous multi-centre surveillance studies conducted in India between 2005 to 2012 [3, 4] Other studies from India have also found these two genotypes as common rotavirus strains causing diarrhoea in children (Table 4). Similar to the surveillance study from 2005 to 2009, an increase in G12 strains, particularly G12P[6], was observed in the southern (Kolenchery, 10.7%) and northern (New Delhi, 12.8%) regions [3] G12 strains were detected first in Philippines in 1987, following which they have been reported from other Asian countries [1, 14, 15] In India, G12 was reported first in 2003 from the eastern region, and since then it has been reported to cause AGE in children from other regions [3, 13, 16, 17] G9, in association with P[4], P[6], and P[8], was detected in 13.2% of the samples, which is higher than reported in the Indian Rotavirus Network Surveillance study from 2005 to 2009 (7.5%) [3]. G9 was first detected in Philadelphia in 1983, and is now the fifth most common G type worldwide [1, 15]. G9 is the most successful among the currently circulating reassortant human rotavirus strains. Similar to G9 rotaviruses, G12 rotavirus strains also exhibit reassortment activity [15]. The emergence of G12 rotaviruses might be similar to the pattern of evolutionary events that led to the emergence of G9 as a dominant human rotavirus genotype. This view is strengthened by the fact that G12 rotavirus strains are increasingly being detected in Argentina, Nepal, Bangladesh, Japan, and other countries globally [15].

The emergence of G3P[8] in our study across southern and northern sites from 2015 onwards emphasizes the rapid temporal changes in circulating rotavirus strains. The circulation of uncommon genotypes, such as G1P[4], G1P[6], G2P[6], G2P[8], G3P[4], G3P[6], G4P[6], G9P[6], G12P[4], and G12P[11], and the high proportion of mixed infections (8%) indicate that the children probably acquire infections from a variety of sources, and could serve as sources of new global strains.

We did not find any association between the severity of diarrhoea and genotypes of the rotavirus, unlike previous studies which reported that children infected with G1 strains had a greater risk of developing severe diarrhoea than children infected with other rotavirus strains [5, 18].

The strengths of this study include the use of a standardized protocol across the sites and laboratory confirmation of rotavirus diarrhoea in a single reference laboratory. The difficulty in extrapolating the data from 7 sites to the whole Indian population can be a potential limitation of the study, but previous studies have shown that data tend to be similar across the country [3].

## Conclusions

To summarize, this study highlights the ongoing high prevalence of rotavirus disease in India and the change in circulating rotavirus strains across sites. The study demonstrates the continued circulation of G9 and G12 strains and the emergence of G3P[8] from 2015 onwards. With new vaccines and the recent introduction of the indigenous oral rotavirus vaccine into the national immunization schedule in nine Indian states by the Government of India, ongoing surveillance is important to interpret the rotavirus epidemiology and evaluation of vaccine effectiveness against a broad range of serotypes, thus assessing the impact of vaccination.

## Additional files

Additional file 1: **Table S1.** List of excluded cases. The list contains details of reasons for site wise exclusion of cases from final analysis. (DOCX 16 kb)

Additional file 2: **Table S2.** Association between rotavirus genotype and severity of diarrhoea. The file contains details of the six common rotavirus genotypes in this study, and their association with severity of diarrhoea in children < 5 years. (DOCX 21 kb)

Additional file 3: **Table S3.** Year wise rotavirus genotype distribution across 7 sites. The file contains details of year wise distribution of rotavirus genotypes in children with acute gastroenteritis in India across 7 sites. (DOCX 19 kb)

Additional file 4: **Table S4.** Rotavirus genotype distribution in north Indian sites. The file contains details of year wise distribution of rotavirus genotypes in the 2 north Indian sites from July 2012 to June 2016. (DOCX 20 kb)

Additional file 5: **Table S5.** Rotavirus genotype distribution in south Indian sites. The file contains details of year wise distribution of rotavirus genotypes in the 5 south Indian sites from July 2012 to June 2016. (DOCX 20 kb)

## Abbreviations

AGE: Acute gastroenteritis
CMC: Christian Medical College, Vellore
EIA: Enzyme immunoassay
RT-PCR: Reverse transcription-polymerase chain reaction
