# Supplementary material for: Rotavirus gastroenteritis in Indian children < 5 years hospitalized for diarrhoea, 2012 to 2016
Source: BMC Public Health. 2019 Jan 15;19:69. doi: 10.1186/s12889-019-6406-0 (PMC6334384; doi:10.1186/s12889-019-6406-0)
Supplement: Supplementary file 1 — Table S1. List of excluded cases. The list contains details of reasons for site wise exclusion of cases from final analysis. (DOCX 16 kb) [file 12889_2019_6406_MOESM1_ESM.docx]

**Table S1:** List of excluded cases

|  | Duration >5 days | No. of episodes <3/24hrs | Age >59 months | Diagnosis absent | Dysentery | Diagnosis absent & duration >5 days | No diarrhoea | Episodes <3/24 hrs & Duration >5 days |
| --- | --- | --- | --- | --- | --- | --- | --- | --- |
| Delhi (237) | 152 | 38 | 4 | 21 | 18 | 4 | - | - |
| Hyderabad (7) | 3 | - | 4 | - | - | - | - | - |
| Kolenchery (210) | 30 | 56 | 6 | 4 | 112 | - | 2 | - |
| Ludhiana (127) | 61 | 22 | - | 13 | 24 | - | 6 | 1 |
| Tirupati (32) | 31 | - | - | - | 1 | - | - | - |
| Trichy (63) | 22 | 28 | - | 11 | 1 | - | - | 1 |
| Vellore (66) | 52 | 1 | - | 2 | 8 | - | 3 | - |
| Total (742) | 351 | 145 | 14 | 51 | 164 | 4 | 11 | 2 |
