# Supplementary material for: Rotavirus gastroenteritis in Indian children < 5 years hospitalized for diarrhoea, 2012 to 2016
Source: BMC Public Health. 2019 Jan 15;19:69. doi: 10.1186/s12889-019-6406-0 (PMC6334384; doi:10.1186/s12889-019-6406-0)
Supplement: Supplementary file 2 — Table S2. Association between rotavirus genotype and severity of diarrhoea. The file contains details of the six common rotavirus genotypes in this study, and their association with severity of diarrhoea in children < 5 years. (DOCX 21 kb) [file 12889_2019_6406_MOESM2_ESM.docx]

**Table S2**: Association between rotavirus genotype and severity of diarrhoea

| **Genotype (no. of positive samples)** | **Vesikari score >10 (Severe diarrhoea)** | **Vesikari score ≤10 (Mild-Moderate diarrhoea)** | **p-value** |
| --- | --- | --- | --- |
| G1P[8] (1002) | 654 (50.3%) | 348 (45.3%) | 0.24 |
| G2P[4] (196) | 122 (9.4%) | 74 (9.6%) | 0.58 |
| G9P[4] (177) | 120 (9.2%) | 57 (7.4%) | 0.32 |
| G12P[6] (105) | 56 (4.3%) | 49 (6.4%) | 0.20 |
| G9P[8] (71) | 41 (3.2%) | 30 (3.9%) | 0.26 |
| G3P[8] (58) | 39 (3%) | 19 (2.5%) | 0.68 |

The six common rotavirus genotypes in this study, and their association with severity of diarrhoea is provided.
