# Supplementary material for: Rotavirus gastroenteritis in Indian children < 5 years hospitalized for diarrhoea, 2012 to 2016
Source: BMC Public Health. 2019 Jan 15;19:69. doi: 10.1186/s12889-019-6406-0 (PMC6334384; doi:10.1186/s12889-019-6406-0)
Supplement: Supplementary file 3 — Table S3. Year wise rotavirus genotype distribution across 7 sites. The file contains details of year wise distribution of rotavirus genotypes in children with acute gastroenteritis in India across 7 sites. (DOCX 19 kb) [file 12889_2019_6406_MOESM3_ESM.docx]

**Table S3:** Year wise rotavirus genotype distribution across 7 sites

| Genotype | July 2012-June 2013 | | July 2013-June 2014 | | July 2014-June 2015 | | July 2015- June 2016 | |
| --- | --- | --- | --- | --- | --- | --- | --- | --- |
|  | **N** | **%** | **N** | **%** | **N** | **%** | **N** | **%** |
| G1P[4] | 6 | 1.5 | 9 | 1.5 | 3 | 0.6 | - | - |
| G1P[6] | 9 | 2.3 | 10 | 1.6 | 6 | 1.1 | 20 | 4.3 |
| G1P[8] | 146 | 37.1 | 366 | 59.9 | 378 | 69.7 | 112 | 24.2 |
| G2P[4] | 66 | 16.8 | 32 | 5.2 | 42 | 7.7 | 56 | 12.1 |
| G2P[6] | 7 | 1.8 | - | - | 4 | 0.7 | 12 | 2.6 |
| G2P[8] | - | - | 1 | 0.2 | - | - | - | - |
| G3P[4] | - | - | - | - | 1 | 0.2 | 3 | 0.6 |
| G3P[6] | - | - | - | - | 1 | 0.2 | - | - |
| G3P[8] | - | - | - | - | 2 | 0.4 | 56 | 12.1 |
| G4P[6] | - | - | - | - | - | - | 1 | 0.2 |
| G9P[4] | 25 | 6.3 | 18 | 2.9 | 30 | 5.5 | 104 | 22.5 |
| G9P[6] | 5 | 1.3 | 7 | 1.1 | 5 | 0.9 | 2 | 0.4 |
| G9P[8] | 13 | 3.3 | 48 | 7.9 | 5 | 0.9 | 5 | 1.1 |
| G10P[11] | 2 | 0.5 | 1 | 0.2 | 2 | 0.4 | 5 | 1.1 |
| G12P[4] | 6 | 1.5 | - | - | - | - | 1 | 0.2 |
| G12P[6] | 40 | 10.2 | 38 | 6.2 | 15 | 2.8 | 12 | 2.6 |
| G12P[8] | 27 | 6.9 | 11 | 1.8 | 6 | 1.1 | 5 | 1.1 |
| G12P[11] | - | - | - | - | - | - | 1 | 0.2 |
| Mixed | 28 | 7.1 | 58 | 9.5 | 21 | 3.9 | 53 | 11.4 |
| Partially typed | 4 | 1.0 | 4 | 0.7 | 5 | 0.9 | 6 | 1.3 |
| Untyped | 10 | 2.5 | 8 | 1.3 | 16 | 3.0 | 9 | 1.9 |
| Total | 394 |  | 611 |  | 542 |  | 463 |  |
