# Supplementary material for: Rotavirus gastroenteritis in Indian children < 5 years hospitalized for diarrhoea, 2012 to 2016
Source: BMC Public Health. 2019 Jan 15;19:69. doi: 10.1186/s12889-019-6406-0 (PMC6334384; doi:10.1186/s12889-019-6406-0)
Supplement: Supplementary file 4 — Table S4. Rotavirus genotype distribution in north Indian sites. The file contains details of year wise distribution of rotavirus genotypes in the 2 north Indian sites from July 2012 to June 2016. (DOCX 20 kb) [file 12889_2019_6406_MOESM4_ESM.docx]

**Table S4**: Rotavirus genotype distribution in north Indian sites

| Genotype | July 2012-June 2013 | | July 2013-June 2014 | | July 2014-June 2015 | | July 2015- June 2016 | | Total | |
| --- | --- | --- | --- | --- | --- | --- | --- | --- | --- | --- |
|  | **N** | **%** | **N** | **%** | **N** | **%** | **N** | **%** | **N** | **%** |
| G1P[4] | 1 | 0.8 | 3 | 1.4 | - | - | - | - | 4 | 0.6 |
| G1P[6] | 2 | 1.5 | 2 | 0.9 | 4 | 2.8 | 16 | 10.8 | 24 | 3.7 |
| G1P[8] | 39 | 29.8 | 115 | 52.3 | 56 | 39.4 | 21 | 14.2 | 231 | 36.0 |
| G2P[4] | 17 | 13.0 | 17 | 7.7 | 28 | 19.7 | 10 | 6.8 | 72 | 11.2 |
| G2P[6] | 2 | 1.5 | - | - | 4 | 2.8 | 11 | 7.4 | 17 | 2.7 |
| G2P[8] | - | - | - | - | - | - | - | - | 0 | 0.0 |
| G3P[4] | - | - | - | - | 1 | 0.7 | 1 | 0.7 | 2 | 0.3 |
| G3P[6] | - | - | - | - | - | - | - | - | 0 | 0.0 |
| G3P[8] | - | - | - | - | 2 | 1.4 | 36 | 24.3 | 38 | 5.9 |
| G4P[6] | - | - | - | - | - | - | - | - | 0 | 0.0 |
| G9P[4] | 20 | 15.3 | 11 | 5.0 | 18 | 12.7 | 24 | 16.2 | 73 | 11.4 |
| G9P[6] | 4 | 3.1 | 5 | 2.3 | 5 | 3.5 | 1 | 0.7 | 15 | 2.3 |
| G9P[8] | 2 | 1.5 | 10 | 4.5 | - | - | 2 | 1.4 | 14 | 2.2 |
| G10P[11] | - | - | - | - | - | - | - | - | 0 | 0.0 |
| G12P[4] | 3 | 2.3 | - | - | - | - | 1 | 0.7 | 4 | 0.6 |
| G12P[6] | 24 | 18.3 | 20 | 9.1 | 7 | 4.9 | 3 | 2.0 | 54 | 8.4 |
| G12P[8] | 1 | 0.8 | 7 | 3.2 | 4 | 2.8 | 1 | 0.7 | 13 | 2.0 |
| G12P[11] | - | - | - | - | - | - | 1 | 0.7 | 1 | 0.2 |
| Mixed | 11 | 8.4 | 28 | 12.7 | 11 | 7.7 | 18 | 12.2 | 68 | 10.6 |
| Partially typed | - | - | 1 | 0.5 | 2 | 1.4 | - | - | 3 | 0.5 |
| Untyped | 5 | 3.8 | 1 | 0.5 | - | - | 2 | 1.4 | 8 | 1.2 |
| Total | 131 |  | 220 |  | 142 |  | 148 |  | 641 |  |
