# Supplementary material for: Rotavirus gastroenteritis in Indian children < 5 years hospitalized for diarrhoea, 2012 to 2016
Source: BMC Public Health. 2019 Jan 15;19:69. doi: 10.1186/s12889-019-6406-0 (PMC6334384; doi:10.1186/s12889-019-6406-0)
Supplement: Supplementary file 5 — Table S5. Rotavirus genotype distribution in south Indian sites. The file contains details of year wise distribution of rotavirus genotypes in the 5 south Indian sites from July 2012 to June 2016. (DOCX 20 kb) [file 12889_2019_6406_MOESM5_ESM.docx]

**Table S5**: Rotavirus genotype distribution in south Indian sites

| Genotype | July 2012-June 2013 | | July 2013-June 2014 | | July 2014-June 2015 | | July 2015- June 2016 | | Total | |
| --- | --- | --- | --- | --- | --- | --- | --- | --- | --- | --- |
|  | **N** | **%** | **N** | **%** | **N** | **%** | **N** | **%** | **N** | **%** |
| G1P[4] | 5 | 1.9 | 6 | 1.5 | 3 | 0.8 | - | - | 14 | 1.0 |
| G1P[6] | 7 | 2.7 | 8 | 2.0 | 2 | 0.5 | 4 | 1.3 | 21 | 1.5 |
| G1P[8] | 107 | 40.7 | 251 | 64.2 | 322 | 80.5 | 91 | 28.9 | 771 | 56.3 |
| G2P[4] | 49 | 18.6 | 15 | 3.8 | 14 | 3.5 | 46 | 14.6 | 124 | 9.1 |
| G2P[6] | 5 | 1.9 | - | - | - | - | 1 | 0.3 | 6 | 0.4 |
| G2P[8] | - | - | 1 | 0.3 | - | - | - | - | 1 | 0.1 |
| G3P[4] | - | - | - | - | - | - | 2 | 0.6 | 2 | 0.1 |
| G3P[6] | - | - | - | - | 1 | 0.3 | - | - | 1 | 0.1 |
| G3P[8] | - | - | - | - | - | - | 20 | 6.3 | 20 | 1.5 |
| G4P[6] | - | - | - | - | - | - | 1 | 0.3 | 1 | 0.1 |
| G9P[4] | 5 | 1.9 | 7 | 1.8 | 12 | 3.0 | 80 | 25.4 | 104 | 7.6 |
| G9P[6] | 1 | 0.4 | 2 | 0.5 | - | - | 1 | 0.3 | 4 | 0.3 |
| G9P[8] | 11 | 4.2 | 38 | 9.7 | 5 | 1.3 | 3 | 1.0 | 57 | 4.2 |
| G10P[11] | 2 | 0.8 | 1 | 0.3 | 2 | 0.5 | 5 | 1.6 | 10 | 0.7 |
| G12P[4] | 3 | 1.1 | - | - | - | - | - | - | 3 | 0.2 |
| G12P[6] | 16 | 6.1 | 18 | 4.6 | 8 | 2.0 | 9 | 2.9 | 51 | 3.7 |
| G12P[8] | 26 | 9.9 | 4 | 1.0 | 2 | 0.5 | 4 | 1.3 | 36 | 2.6 |
| G12P[11] | - | - | - | - | - | - | - | - | 0 | 0.0 |
| Mixed | 17 | 6.5 | 30 | 7.7 | 10 | 2.5 | 34 | 10.8 | 91 | 6.6 |
| Partially typed | 4 | 1.5 | 3 | 0.8 | 4 | 1.0 | 7 | 2.2 | 18 | 1.3 |
| Untyped | 5 | 1.9 | 7 | 1.8 | 15 | 3.8 | 7 | 2.2 | 34 | 2.5 |
| Total | 263 |  | 391 |  | 400 |  | 315 |  | 1369 |  |
